# Vaginal Use of Ibuprofen Isobutanolammonium (Ginenorm): Efficacy, Tolerability, and Pharmacokinetic Data: A Review of Available Data

**DOI:** 10.5402/2012/673131

**Published:** 2012-07-09

**Authors:** Massimo Milani, Piero Iacobelli

**Affiliations:** ^1^Solo Practice, Via A. Nota 18, 20126 Milan, Italy; ^2^Department of Gynaecology Ospedale Fatebenefratelli, Napoli, Italy

## Abstract

Vaginal infection and inflammation with or without vulvar involvement are very common gynecologicaly clinical conditions associated with morbidity and reduced quality of life. Vaginal infections are commonly treated with causal antimicrobial treatments. In addition to specific antimicrobial treatment, anti-inflammatory therapy, both systemic or topical (vaginal douche), could be useful in the integrated treatment approach of these conditions reducing symptoms and speeding up the recovery in vulvovaginitis. Ibuprofen is a well-known effective and well-tolerated anti-COX (anti-COX1 and COX2) compound. In addition, several in vitro studies suggest that Ibuprofen shares antimicrobial and antifungal activities. Ibuprofen isobutanolammonium (Ib-isb) (Ginenorm) is a soluble salt from formulation suitable for external and intravaginal use. This salt completely dissociates in aqueous solution. Ib-isob is available in sachet and vaginal douche pharmaceutical formulations. Clinical efficacy of Ib-isob has been documented in 10 clinical studies (6 controlled and 4 open trials) which have enrolled in total 399 women with vulvovaginitis. The six controlled clinical trials were performed both versus placebo (2 studies) or versus active comparators such as benzydamine. In these studied, Ib-Isb has been used in general for 7 consecutive days with a twice application daily regimen at the dose of 1 g per application. Topical application of Ib-isob induced a marked and rapid reduction in signs (erythema, oedema) and symptoms (itching and burning sensation) of vulvovaginitis. In head-to-head studies carried out in comparison with other topical products, Ib-isob induced a more rapid reduction in both subjective and objective symptoms. In particular a remarkable significant improvement of all the symptoms has been observed in the group of patients treated with Ib-isob in comparison with women receiving benzydamine. The clinical data available for Ib-isob confirm that this salt, specifically developed for gynecological use, is effective and well tolerated in vulvovaginal inflammation conditions. Efficacy of Ib-isob was greater in comparison with commonly used products. Ibuprofen-isob may be considered a useful and effective tool for the topical treatment of nonspecific vaginal diseases.

## 1. Introduction

Vaginal infection and inflammation with or without vulvar involvement are very common gynecological clinical conditions associated with morbidity and reduced quality of life [1]. Vulvovaginitis is characterized by inflammation of vaginal mucosa with vulval involvement. Vulvovaginitis could have both noninfective (allergic forms, hormonal alteration, concomitant systemic, or topic pharmacological treatments) and infective causes (fungal, bacterial or protozoa). Burning, itching, and pain sensation are common symptoms of vulvovaginitis. The most common sign of vaginal infection is vaginal discharge. Vaginal discharge is highly variable in quality and quantity among different individuals, and even in the same individual during different periods of life. Symptoms of vaginitis, account for over 10 million office visits per year [2] in USAand frequently leading to self-diagnosis and therapy with the use of over-the-counter products. Vaginitis is responsible for >10% of visits made to providers of women's health care [3]. Vaginal discharge is most commonly caused by infection with sexually transmitted organisms or increased colonization by different facultative pathogenic microorganisms (i.e., Gardnerella vaginalis) [4]. Most common in women with a vaginal infection is bacterial vaginosis (40–50% of cases), followed by vulvovaginal candidosis (20–25%), and then trichomoniasis (15–20%) [5]. If infection is suspected as the primary cause, a sample of the vaginal discharge should be taken and examined microscopically. Candidiasis, trichomonas, and bacterial vaginosis are the most common forms of vaginal infections [6]. Noninfectious causes of vaginal discharge are less frequent but anyway they are a relevant part of vaginitis [7] especially in the young woman. Although vaginal infections are the most common cause, other considerations include cervicitis, a normal vaginal discharge, foreign-body vaginitis, contact vaginitis, atrophic vaginitis, and desquamative inflammatory vaginitis [8]. The medical history and examination are an important source of clues to the underlying diagnosis. However, making a definitive diagnosis requires skilful performance of office laboratory procedures, including the vaginal pool wet mount examination, determination of the vaginal pH, and the whiff test [9]. Vaginal and cervical cultures, nucleic acid tests, and point-of-care tests are available and may be required in selected patients. Once a specific diagnosis is made, effective therapy can be prescribed. Candida vaginitis is generally treated with either the vaginal administration of an imidazole or triazole antifungal agents or the prescription of oral fluconazole [10]. Oral nitroimidazole agents, metronidazole or tinidazole, are the only effective treatments for trichomoniasis commonly used [11]. Bacterial vaginosis, which has been linked to important gynecologic and pregnancy complications, can be treated with an available oral or topical agents containing either a nitroimidazole or clindamycin [12]. In addition to specific causal therapy (antibacterial or antifungal products), vaginitis often requires a symptomatic treatment in order to reduce signs and symptoms associated with the clinical condition [13] such as pain burning and itching sensations. Oral anti-inflammatory agents could be used in these conditions but their use could be associated with the common side effects of this class of drugs [14]. A vaginal formulation of ibuprofen (douche and sachet) has been available with the indication of symptomatic treatment of vulvovaginitis conditions. Topical application of an anti-inflammatory agent could have the advantage to increase locally the therapeutic effect and at the same time to reduce the systemic exposure reducing the risk of side effects [15].

## 2. Ibuprofen: Pharmacodynamic ****Characteristics

Cyclooxygenase (COX) is the pivotal enzyme in prostaglandin biosynthesis. It exists in two isoforms, constitutive COX-1 (responsible for physiological functions) and inducible COX-2 (involved in inflammation). Inhibition of COX explains both the therapeutic effects (inhibition of COX-2) and side effects (inhibition of COX-1) of non-steroidal anti-inflammatory drugs (NSAIDs) [16]. Ibuprofen is a well-known effective, safe and well-tolerated anti-COX1 compound [17] with anti-inflammatory, antipyretic, and antidolorifico activities [18]. The action mechanism of Ibuprofen, common to the other nonsteroid antiphlogistic drugs is due to the inhibition of prostaglandin synthesis [19]. Ibuprofen has a good safety profile comparable with paracetamol [20]. Its analgesic activity is linked to its anti-inflammatory effects and is related to reduction in the ex vivo production in blood of cyclooxygenase (COX)-1 and COX-2 derived prostanoids. Nonsteroidal anti-inflammatory drugs such as ibuprofen work by inhibiting the enzyme cyclooxygenase (COX), which converts arachidonic acid to prostaglandin H_2_ (PGH_2_). PGH_2_, in turn, is converted by other enzymes to several other prostaglandins (which are mediators of pain, inflammation, and fever) and to thromboxane A_2_ (which stimulates platelet aggregation, leading to the formation of blood clots). Like aspirin, indomethacin, and all other NSAIDs, ibuprofen is considered a nonselective COX inhibitor; that is, it inhibits two isoforms of cyclooxygenase, *COX*-1 and *COX*-2. The analgesic, antipyretic, and anti-inflammatory activity of NSAIDs appears to be achieved mainly through inhibition of COX-2, whereas inhibition of COX-1 would be responsible for unwanted effects on platelet aggregation and the gastrointestinal tract. However, the role of the individual COX isoforms in the analgesic, anti-inflammatory, and gastric damage effects of NSAIDs is uncertain and different compounds cause different degrees of analgesia and gastric damage. Higher doses of ibuprofen are employed long-term for the treatment of rheumatic and other more severe musculoskeletal conditions [21]. Recent evidence from large-scale clinical trials with the newer coxibs, where ibuprofen was as a comparator, has confirmed earlier studies which have shown that ibuprofen has comparable therapeutic benefits with coxibs and other NSAIDs [22]. The particular active anti-inflammatory action of Ibuprofen has permitted the creation of a preparation for largely topical use in inflammatory conditions of the osteoarticular and muscular apparatus [23]. Also formulation for vaginal applications (sachet for vaginal douche) is now available. Beside anti-inflammatory action, several in vitro studies suggest that Ibuprofen demonstrated antimicrobial and antifungal activities. These additional properties of Ibuprofen suggest that the vaginal use of this product could be particular useful in condition such as vulvovaginitis.

## 3. Ibuprofen: Antimicrobial Activity

In vitro studies with Ibuprofen have demonstrated that this molecule, beside its anti-inflammatory activity, shares also an antimicrobial action [24]. The studies of Drago et al. [25, 26] showed that ibuprofen has a significant antimicrobial activity especially against Gram-negative strains, *Gardnerella vaginalis* but also against *Candida albicans*. In addition Ibuprofen interferes with the adhesion of *Gardnerella vaginalis* and *Candida albicans* to the vaginal epithelium. Other in vitro studies show that Ibuprofen seems to have a synergic activity with both antimycotic and antimicrobial properties on germs isolated by vaginal samples, mostly versus *Candida albicans* and *Gardnerella vaginalis*. However, this mechanism is not yet clear and these data require further confirmation. In addition, it has been shown that at subclinical concentrations Ibuprofen isobuthanolammonium seems to be able to interfere with the adhesion of *Gardnerella vaginalis* to the epithelial cells of the vagina, one of the main pathogenetic factors of this microorganism. In combination with econazole, Ibuprofen isobutanolammonium appeared to be able to interfere with *Candida albicans* tube formation, a pathogenetic factor that allows the microorganism to penetrate in the mucous cells. More recently, Drago et al. [27] investigated the presence of synergistic activity of econazole, and ibuprofen isobuthanolammonium against 310 different vaginal isolates, by using the microdilution broth assay to test in vitro antimicrobial activity and the effect of the two drugs on phagocytosis and intramacrophagic cellular killing of mouse peritoneal macrophages. The effect of subinhibitory concentrations of econazole/ibuprofen isobuthanolammonium combination on *Candida albicans* germ-tube formation was also evaluated. The in vitro antifungal activity of econazole was notably improved by addition of ibuprofen isobuthanolammonium. Macrophage killing of *C. albicans* was significantly increased by the two drugs and also germ-tube formation was significantly affected. The investigators concluded that the addition of ibuprofen isobuthanolammonium to econazole provides better in vitro antifungal activity. These data suggest that vaginal infections caused by *Candida albicans* treated with econazole could be improved by addition of ibuprofen isobuthanolammonium. All these data support the evidence that ibuprofen has also an antimicrobial activity against Gram-positive, Gram-negative, and fungal microorganisms. These characteristics could offer a strong rationale for the development of a vaginal formulation of ibuprofen to be used in inflammatory/infective conditions involving vulva and vagina.

## 4. Ibuprofen Isobutanolammonium for Topical ****Vaginal Use: Pharmacological Characteristics

Ibuprofen isobutanolammonium (Ib-isb) (Ginenorm) is a soluble salt form formulation suitable for external and intravaginal use. Ib-isb is a soluble salt of ibuprofen and chemically corresponds to the following formula: 2-amino-2-methyl-1-propanol-*ε*-methyl-4-(2-methylpropyl) benzene acetate. This salt completely dissociates in aqueous solution. Ib-isob is available in sachet and vaginal douche. The vaginal administration of Ginenorm does not lead to a large absorption of ibuprofen across the vaginal mucus membrane. After vaginal application, ibuprofen plasma levels are extremely low (0.2–0.3 *μ*g/mL at the second hour from administration). The passage of ibuprofen into systemic circulation in unmodified form can eventually reach a very low quota. In fact, absorption across the vaginal mucus membrane should be considered almost absent. Ib-isb does not modify vaginal pH, the activity of the bacillus Doderlein and consequently vaginal ecosystem. Therefore vaginal use of this compound is not associated with potential dangerous modifications of the vaginal ecosystem. The possibility to use a topical formulation of ibuprofen improved in a significant manner, the therapeutic index in comparison with the systemic route of administration. 

## 5. Ibuprofen Isobutanolammonium—Clinical ****Efficacy Data: A Review

Clinical efficacy of Ib-isob (Ginenorm) has been documented in a total 10 clinical studies which have enrolled a total 399 women with vulvovaginitis [28]. The clinical plan of vaginal use of Ib-isb was designed to evaluate the anti-inflammatory activity and to assess the local tolerability. The clinical plan has foreseen comparisons with placebo (2 trials) with active comparator (benzydamine) (4 trials) and finally 4 open, not controlled studies. Women enrolled in these trials in general suffered from vulvitis or vaginitis only or vulvovaginitis. In these studies, Ib-Isb has been used for 7 consecutive days with a twice application per day regimen at the dose of 1 g per application. All these studies showed that topical application of Ib-isob induced a marked and rapid reduction in signs (erythema, oedema) and symptoms (burning and itching) of vulvovaginitis.

### 5.1. Efficacy versus Placebo

**Figure 1:**
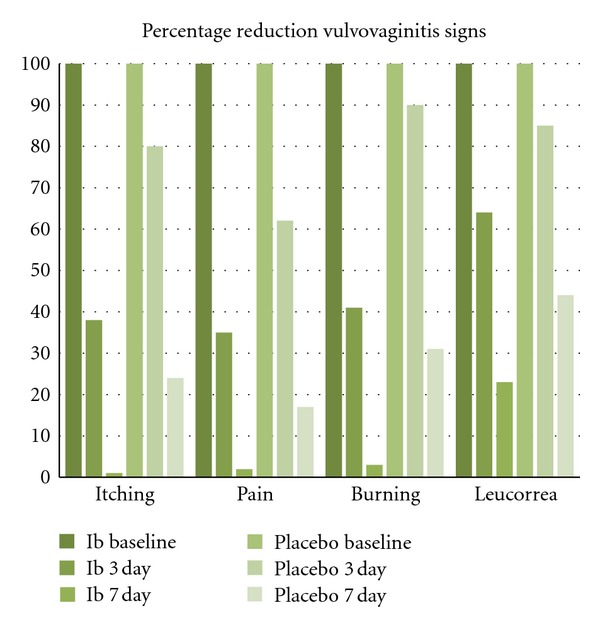
Efficacy of Ginenorm in comparison with placebo: percentage reduction at day 7.

A total of 2 studies had evaluate the efficacy of Ib-isob in comparison with placebo in 70 women with vulvovaginitis. In these studies, the treatment with Ib-isb induced a significant reduction of itching, pain, burning sensation, leucorrea, erythema, and redness of vaginal mucosa. This reduction was statistically significantly greater in comparison with placebo and the clinical effect was observed after 3 days of treatment.  Figure 1 shows the percentage reduction in signs and symptoms in active and placebo treated women at the end of 7 days of treatment. In particular itching and pain sensation disappeared after 7 day of treatment in women treated with Ib-isob.

### 5.2. Efficacy versus Benzydamine

**Figure 2:**
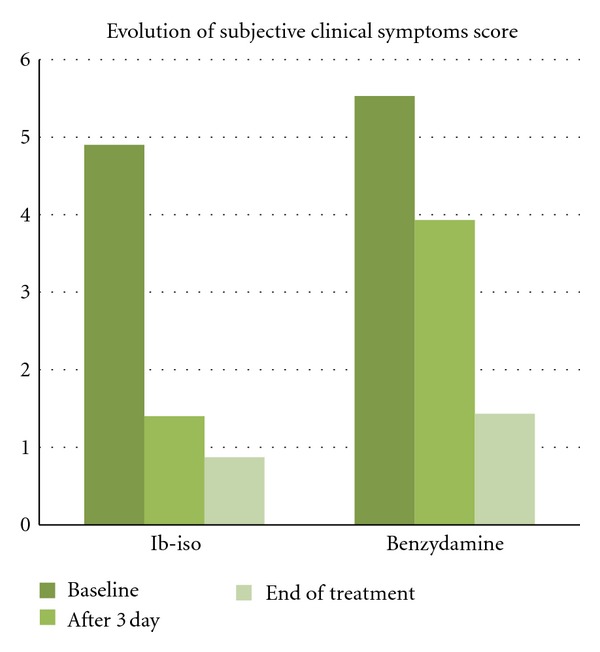
Overall trend of subjective symptom score in women with vulvovaginitis (from Pullè).

**Figure 3:**
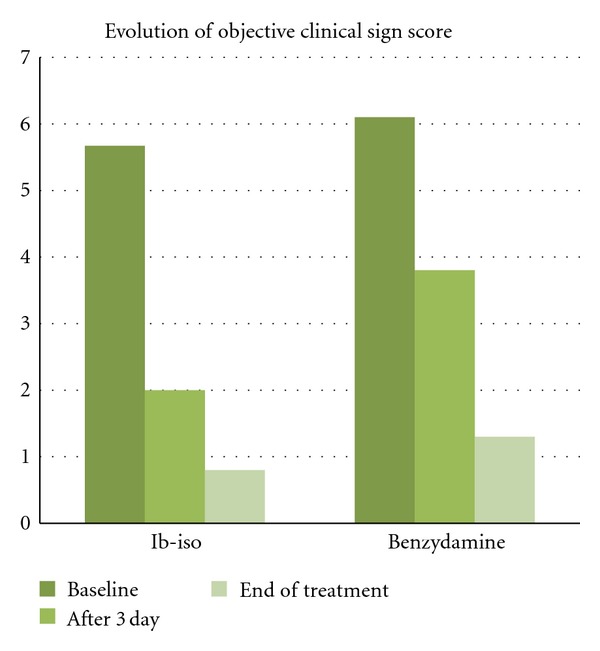
Overall trend of objective symptom score in women with vulvovaginitis (from Pullè).

 In head-to-head studies (a total of 8) carried out in comparison with other topical products such as benzydamine, the efficacy of Ib-isob induced a more rapid reduction in subjective and objective symptoms. In these studies, Ginenorm appears to be efficacious in improving symptoms (itching, burning, leukorrhea, redness) in phlogistic forms of the vulvovaginal apparatus such as vaginitis-vulvovaginitis of mixed genesis. The administration of Ibuprofen, in the form of vaginal douches in patients affected by phlogistic vulvovaginal disorders demonstrated a marked therapeutic effect on the body of symptoms associated with vaginitis. Pullè and Sturlese [29] have studied Ib-Isb on 30 female patients (average age: 40.5 ± 11 years, age range 19–65), affected by vulval and/or vaginal phlogosis with a prevalence of uterine fibromyomatosis and metrorrhagia of a varied nature. Sixteen patients were affected with vulvitis and 14 with vulvovaginitis. Fifteen patients were assigned to the treatment with Ibuprofen isobutanolammonium and 15 to the treatment with Benzydamine hydrochloride. Both the subjective symptoms (burning and itching) and the objective symptoms (erythema, oedema, exudation) showed a marked reduction as early as the third day of treatment and the comparison between the score at the start of treatment and on day three of the treatment was highly significant in both study groups (*P* < 0.01). This improvement was, however, more marked in the group of patients treated with ibuprofen isobutanolammonium, where a more rapid reduction of both the subjective and objective symptoms was observed on the third day. The clinical efficacy trend was confirmed at the end of the study, on the seventh day. In this study, Ib-isb showed at the end of treatment period a greater effect in reducing both subjective (burning and itching) and objective (erythema and oedema) clinical signs of vulvovaginitis (Figures 2 and 3).

## 6. Conclusion

Vulvovaginitis is a common gynecological condition [30]. This term identifies in general an inflammatory state of vaginal mucosa often associated with alteration of vaginal ecosystem. Vaginitis and vulvovaginitis could have an infective or noninfective etiology [31]. In an adult woman's reproductive years, the normal bacterial flora contains numerous microorganisms, including aerobic and anaerobic Gram-positive and Gram-negative bacteria. *Lactobacillus * and *Corynebacterium* predominate over other bacteria such as *Streptococcus*, *Bacteroides*, *Staphylococcus*, and * Peptostreptococcus.* Both *Lactobacillus* and *Corynebacterium* produce lactic and acetic acid from glycogen, thus maintaining the low vaginal pH [32]. Additional bacteria are kept in check by the acid-producing bacteria and are rarely pathogenic, but they may become pathogenic if the environmental balance is affected. The skin of the vulva is sensitive to the vaginal environment and hormonal, metabolic, and allergic influences [33]. It is composed of stratified squamous epithelium that contains hair follicles, sebaceous sweat glands, and apocrine glands. Vulvovaginitis is the most common gynecologic condition seen by practitioners and gynecologists [34]. The term *vulvovaginitis* is a broad definition that categorizes many vaginal infections as vulvovaginitis because this two conditions are interrelated. Discharge, burning, and itching are the most common symptoms, accompanied by signs of vulvar irritation such as erythema and excoriation of the vulvar skin. Ibuprofen already present in the market for many years, is an analgesic anti-inflammatory drug, also with a marked antipyretic activity; the drug is a powerful inhibitor of prostaglandin synthesis and exerts its activity by inhibiting its synthesis peripherally. Ibuprofen finds therapeutic application as an antirheumatic (osteoarthrosis, fibrositis, tenosynovitis, myositis, etc.), as an analgesic, (in traumatology, obstetrics, surgery, etc.), and for topical use in cream form in treating contusions, twists, myalgia, arthralgia, lumbago, and so forth. Ibuprofen isobutanolammonium can be considered as a topical formulation of ibuprofen suitable for application in the gynecological field. Preclinical and clinical studies demonstrated that Ib-isb is extremely well tolerated when administered by intravaginal irrigation in the various animal species, even at doses higher than those foreseen in therapy. According to the clinical documentation available and according to the data coming from 10 controlled clinical trials, Ginenorm appears to be efficacious in improving symptoms in phlogistic forms of the vulvovaginal apparatus such as vaginitis-vulvovaginitis of mixed genesis. Its efficacy was assessed in open trials, but especially in controlled studies, both against placebo and against well-known topical anti-inflammatory preparation for gynaecological use (i.e., benzydamine). Vaginal application of Ib-isb is associated with a rapid and marked clinical improvement of signs and symptoms of vulvovaginitis. In vaginal candidosis, Ib-isb could have a synergistic antimycotic effect in association with econazole. Ibuprofen-isob may be considered a useful and effective tool for the topical treatment of nonspecific vaginal diseases.
